# Preparation and characterization of superior hydrophilic PVDF/DA membranes by the self-polymerization approach of dopamine

**DOI:** 10.3389/fchem.2023.1162348

**Published:** 2023-03-30

**Authors:** Shaofeng Li, Meilin Zhang, Jian Sun, Jianping Sun, Ying Wang

**Affiliations:** ^1^ School of Materials and Environmental Engineering, Shenzhen Polytechnic, Shenzhen, Guangdong, China; ^2^ School of Municipal and Environmental Engineering, Shenyang Jianzhu University, Shenyang, Liaoning, China; ^3^ State Key Joint Laboratory of Environment Simulation and Pollution Control, School of Environment, Tsinghua University, Beijing, China

**Keywords:** PVDF/DA membrane, hydrophilicity, response surface methodology, EPS, biodiversity

## Abstract

Polyvinylidene fluoride (PVDF) membranes are favored for their excellent properties. However, the inherent strong hydrophobicity of PVDF membranes limits their development in the field of water treatment. The objective of this study was to improve the performance of PVDF membranes using the self-polymerization, strong adhesion properties, and biocompatible effects of dopamine (DA). The PVDF/DA membrane modification conditions were simulated and optimized using response surface methodology (RSM), and the experimental design was used to investigate three main parameters. The results showed that the DA solution concentration of 1.65 g/L, the coating time of 4.5 h, the post-treatment temperature of 25°C, the contact angle decreased from 69° to 33.9°, and the pure water flux on the PVDF/DA membrane was higher than that on the original membrane. The absolute value of the relative error between the actual and predicted values is only 3.36 %. In the MBR parallel comparison test, compared with the PVDF/DA membrane, the total amount of extracellular polymers (EPS) of the PVDF membrane increased by 1.46 times and the polysaccharide increased by 1.56 times, which further showed that the PVDF/DA modified membrane had the excellent anti-pollution ability. Through Alpha diversity analysis, the biodiversity detected on PVDF/DA membranes was higher than that of PVDF membranes, which further proved its good bio-adhesion ability. These findings could offer a reference for the hydrophilicity, antifouling, and stability of PVDF/DA membranes, which would establish the foundation for the comprehensive applications in MBR.

## 1 Introduction

At present, membrane technology has a broad application prospect in wastewater treatment. Membrane materials with high water flux, high strength, and antifouling properties are key to enhancing MBR treatment, and PVDF has become the main membrane material for microfiltration and ultrafiltration in wastewater treatment because of its easy operational performance, good thermal stability, and excellent chemical stability (Ayyaru and Ahn, 2017; Wang J. et al., 2019; Xiao et al., 2019). Due to the strong hydrophobicity of PVDF, it leads to low water flux after membrane formation, serious membrane contamination problems, and reduced membrane lifetime, which greatly limit the application of membrane bioreactors (Meng et al., 2017; Su and Zhang, 2018; Tang et al., 2019). Therefore, it is important to modify the membranes with necessary hydrophilic modifications to improve the overall performance (Sun et al., 2013; Zhao et al., 2015; Al Mayyahi, 2018). Many hydrophilic modification methods have been becoming hot, and such modification techniques mainly include surface grafting (Sun and Feng, 2017; Dong et al., 2018; Wang et al., 2023), hybrid modification (Qiu et al., 2018; Muchtar et al., 2019; Yu et al., 2022), and surface coating (Zeng et al., 2016; Guo et al., 2017; Zhang et al., 2023). Compared with other methods, surface coating is not only easy to operate but can also be used for large-scale industrial applications.

Traditional surface modification methods are time-consuming or involve complex reaction processes and are largely limited by the surface properties of the substrate. Therefore, developing an effective and versatile strategy remains an unsolved problem in the surface modification of various materials. Lee et al. (2007) proposed a facile method for surface modification of materials by dip coating in dopamine solution, a protein with superior adhesion properties secreted by the marine mussel, and the structure of dopamine is similar to that of the mussel adhesion protein (Cheng et al., 2016; Xue et al., 2017; Wang Z. et al., 2019). In general, dopamine surface modification is simple to operate, gentle and controllable, and environmentally friendly, so this modification method is expected to be used in a wide range of applications (Zhang et al., 2019; Li et al., 2020).

Early studies have shown that dopamine is beneficial to inhibit membrane contamination due to its high hydrophilicity rich in amine and hydroxyl groups, and DA coating has been developed as a general method for surface modification of materials (Yang et al., 2019; Huang et al., 2020). Xiang et al. (2015) immersed the PVDF membrane in a Tris-HCl solution of 2 g/L dopamine (pH = 8.5), and the contact angle of the modified membrane decreased from (118 ± 1.5)° to (53 ± 2.3)°, showing the excellent anti-fouling ability to proteins, and quickly recovered the flux after membrane cleaning. Zin et al. (2019) dissolved different concentrations of DA and polyethyleneimine (PEI) in Tris-HCl buffer solution, soaked the PVDF membrane in it, and found that the modified membrane had good wettability, was highly stable in the pH range of 2–14, and greatly reduced the irreversible pollution of oily wastewater. Mulyati et al. (2020) investigated the effect of two-step dopamine–dopamine modification on polyethersulfone (PES)-based ultrafiltration membranes. The results showed that the higher dopamine load improved the pure water flux by more than three times, hydrophilicity, antifouling, and anti-UV performance. It is clear that the hydrophilicity of the membrane surface has a great influence on membrane contamination, but most studies have focused on the effect of hydrophilicity, and less attention has been paid to the operation of PVDF/DA membranes with improved hydrophilicity in MBR.

In this study, the RSM was used for the first time to model and study the modification process of PVDF/DA membranes. Three main parameters of the modification process were investigated, and based on the experimental data, a significantly effective model was designed using the Box–Behnken method to predict the actual parameters. In addition, the parameters were optimized by considering the appropriate response regions. We chose dopamine for surface modification. By simply immersing the membrane in an alkaline dopamine solution, DA coating will grow on the membrane surface due to oxidative polymerization, and it can significantly improve the hydrophilicity, anti-pollution properties, and biodiversity of the modified membrane, making it suitable for use in membrane bioreactors.

## 2 Experiment

### 2.1 Materials

The PVDF microfiltration membrane (0.22 μm) was purchased from Zhongke Ruiyang Membrane Technology Co., Ltd. (Shunyi district, Beijing city, China). Dopamine hydrochloride and Tris (hydroxymethyl) aminomethane-hydrochloric acid (Tris-HCl) were purchased from Aladdin Bio-Chem Technology Co., Ltd. (Shanghai city, China). The chemicals, isopropanol, glucose, starch, peptone, sodium bicarbonate, dipotassium hydrogen phosphate, and hydrochloric acid (HCl) were obtained from Comio Chemical Reagent Co., Ltd. (Tianjin city, China). Deionized (DI) water was provided by the Milli-Q Water Purification System.

### 2.2 Preparation of the PVDF/DA modified membrane

**FIGURE 1:**
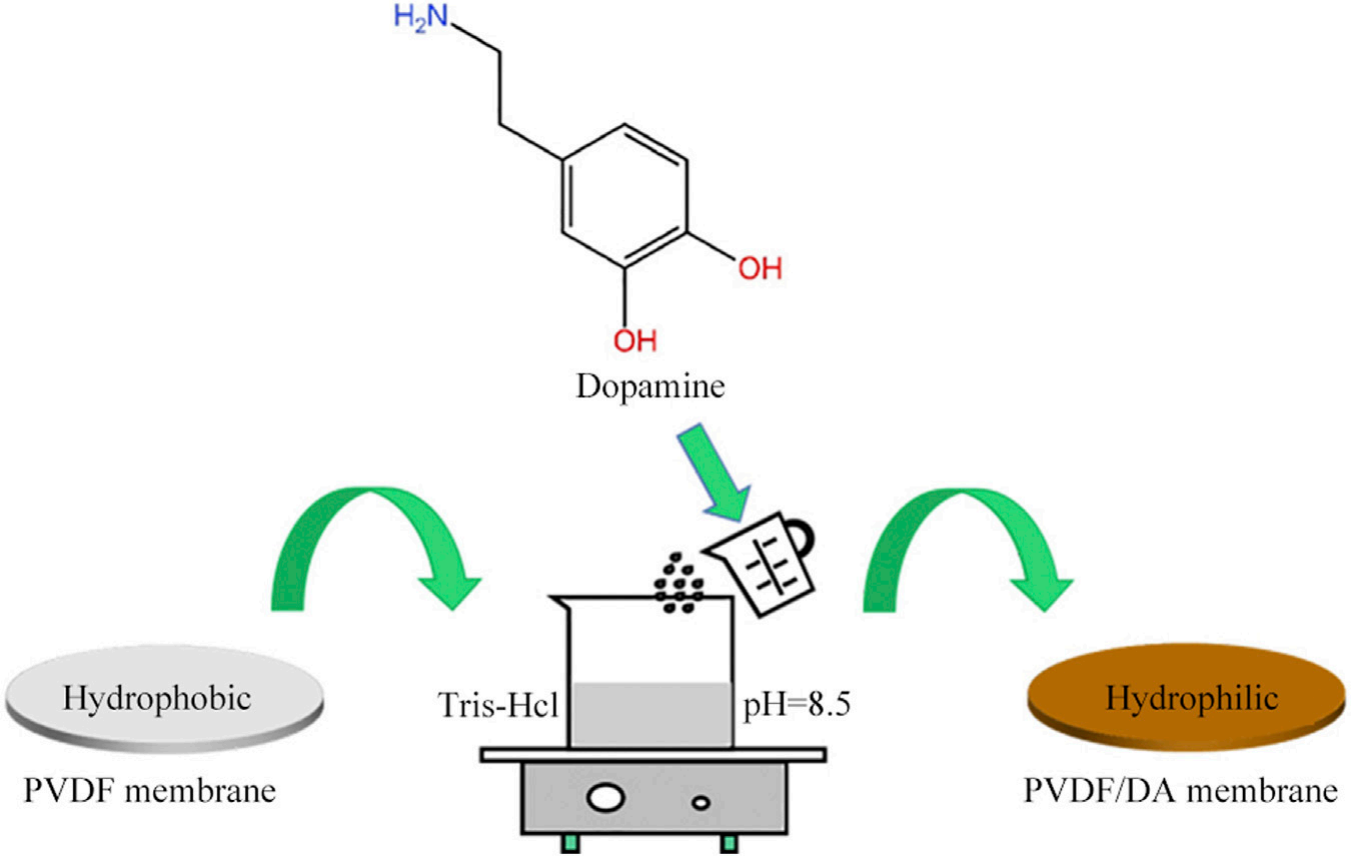
Procedure of the dopamine-modified PVDF membrane.

First, 0.01 mol/L Tris-HCl buffer solution with pH = 8.5 was prepared, and a certain amount of DA was dissolved to form a DA solution. The PVDF membrane was soaked in isopropyl alcohol for 2 h to remove the impurities in the membrane pores and then soaked in distilled water for 12 h to be used. The pretreated PVDF membrane was immersed in DA solution and kept in a shaker (SHA-C, Hangzhou Jingfei Instrument Technology Co., China) at a constant temperature at 120 r/min oscillation. At the end of the self-polymerization reaction process, the membrane material was taken out, rinsed with deionized water, and kept in a vacuum drying oven (DHG-9140A, Shanghai Precision Scientific Instrument Co., Ltd. China), and the PVDF/DA membrane was obtained after drying. The illustration of the membrane fabrication method is shown in Figure 1.

### 2.3 Membrane characterization

All membranes were washed with deionized water and dried before characterization. The surface roughness of the membrane was characterized by atomic force microscopy (AFM, Dimension Icon, Bruker Co., Germany). The chemical composition of the membrane surface was characterized by X-ray photoelectron spectroscopy (XPS, Nexsa, Thermo Fisher Scientific, United States). The hydrophilicity of the membrane surface was measured using a contact angle goniometer (Model No. 250, Rame-Hart, United States). The samples were cut into approximately 5 × 1 cm for contact angle testing. To reduce the experimental error, each sample was tested for at least five points and the average value was taken.

In this experiment, microorganisms on biofilms were detected, DNA extraction quality was detected by agarose gel electrophoresis, and DNA was quantified using a ultraviolet spectrophotometer. The amplification primers were F: 5′-GTGYCAGCMGCCGCGGGTAA-3′ and R: 5′-GGACTACHVGGGTWTCTAAT-3'. Alpha diversity analysis was performed using the Agilent 2100 Bioanalyzer and Illumina’s Library Quantitation Kit, and 2 × 250bp paired-end sequencing, using the NovaSeq 6000 sequencer.

### 2.4 Permeability performance

The pure water flux was measured using a laboratory-made membrane module device, which was composed of two membranes placed symmetrically and prepared from plexiglass, with an inner diameter and an outer diameter of 5.5 and 6.5 cm, respectively, and a total area of 60.5 cm^2^. First, the membrane is pressed with deionized water at 0.10 MPa for 30 min, and after the value is stable, the pure water flux is calculated according to the equation.

$$J=\frac{V}{At}$$ (1)

where *J* is the membrane flux mL/(cm^2^. h), *V* is the water output mL, *A* is the effective membrane filtration area cm^2^, and *t* is the time h.

### 2.5 Stability assessment

To ensure the long-term separation performance of PVDF/DA modified membranes in MBR, the absorbance intensity of DA in the eluent was characterized using an ultraviolet-visible spectrophotometer (UV-7504(A), Shanghai Huyueming Scientific Instrument Co., Ltd. China) to determine the stability of DA coating. The PVDF/DA modified membrane was immersed in 100 mL of deionized water and placed in an ultrasonic cleaner (100 kHz, 25°C) for 30 min, and the concentration of DA in the eluate was measured to characterize the physical stability of the modified membrane. The PVDF/DA modified membrane was immersed in 100 mL of four different conditions of the solution [strong acid (pH = 1), neutral solution (pH = 7), strong base (pH = 13), and acetone (1 + 1)], placed in a shaker and shaken for 2 h (temperature 25°C, 120 r/min), and the concentration of DA in the eluate was determined to characterize the chemical stability of the modified membrane.

### 2.6 MBR experiment and analytical methods

**FIGURE 2:**
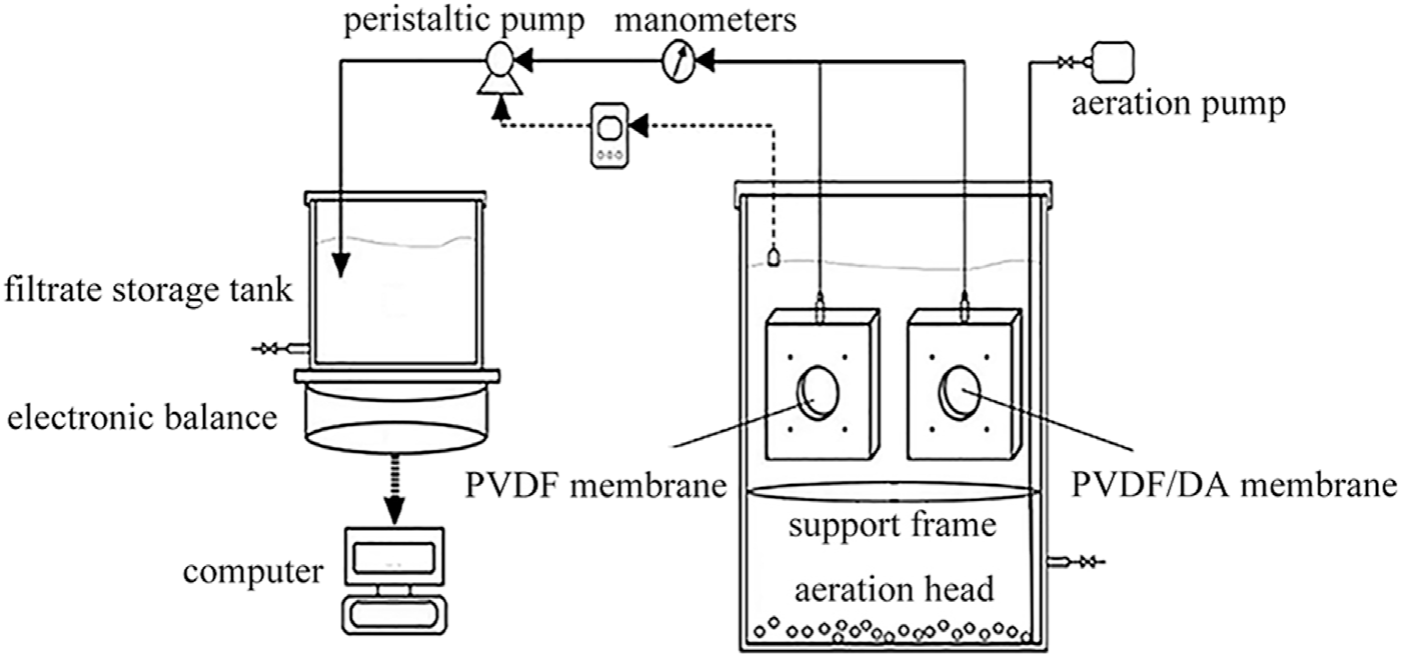
Schematic diagram of the MBR experimental device.

The activated sludge comes from the second sedimentation tank of Shenzhen Deep water Bright water Environment Co., China. It is prepared by artificial water distribution, which is made of glucose, starch, sodium bicarbonate, peptone, dipotassium hydrogen phosphate, *etc.* An integrated immersion membrane bioreactor is adopted, and the intermittent water supply test device is shown in Figure 2. The membrane module consists of two membranes placed symmetrically with a total area of 60.5 cm^2^. During the whole experimental process, no sludge discharge is carried out, and the hydraulic residence time is maintained at a reasonable level by adjusting the pressure of the peristaltic pump, and the pump-stop time ratio is 8: 2. During the hanging period, microorganisms slowly proliferate on the membrane, gradually changing from a yellowish color to a yellowish brown color. COD is measured by using the potassium dichromate method. pH values and DO are measured by using a dissolved oxygen analyzer (JPB-607A, Shanghai Lei Magnetic Instrument Co., China). MLSS is measured by the gravimetric method. EPS was determined by 60°C thermal extractions, and proteins, polysaccharides, and DNA were determined by Folin–phenol, phenol–sulfuric acid, and diphenylamine chromogenic methods, respectively (Drews, 2010).

## 3 Results and discussion

### 3.1 Single-factor experiment for hydrophilicity and membrane flux

#### 3.1.1 Effect of the DA solution concentration

**FIGURE 3:**
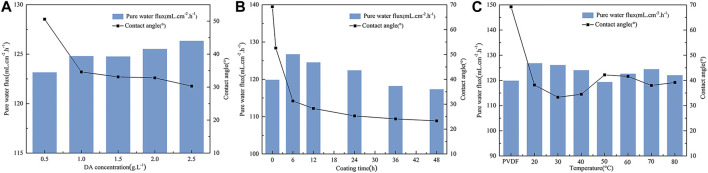
Effect of on contact angle and pure water flux of the PVDF membrane. **(A)** DA solution concentration, **(B)** coating time, and **(C)** temperature.

The effects of different DA solution concentrations on the PVDF membrane contact angle and pure water flux are shown in Figure 3A. It can be seen from the figure that with the increase of the concentration of DA solution, the contact angle of the PVDF membrane surface decreases and the pure water flux rises because dopamine self-polymerizes, and there are key components with strong adhesion in the formed polymer, such as catechol groups and amino functional groups, which greatly improves the hydrophilicity of the membrane surface and is more conducive to the attachment and growth of biofilms on the PVDF membrane surface.

#### 3.1.2 Effect of coating time

The hydrophilicity and pure water flux of the modified PVDF membrane surface were tested at different coating times, and the results are shown in Figure 3B. As can be seen from the figure, the contact angle of the PVDF membrane surface before modification was 69°; with the extension of dopamine coating time, the membrane surface contact angle shows a rapid downward trend, and after about 1 h of the PVDF/DA modified membrane surface contact angle dropping to 52.6°, it finally maintains at 23.3°. It is indicated that the hydrophilicity of the PVDF membrane surface is gradually enhanced, and the pure water flux first rises and then declines, which indicates that the longer the coating time, the thicker and denser the polydopamine layer coated on the membrane surface, resulting in the membrane surface becoming rougher so that hydrophilicity is better. During this period, the longer the coating time, the worse the pure water flux, so the coating coated on the membrane surface cannot be very thick.

#### 3.1.3 Effect of temperature

It can be seen from Figure 3C that compared with the original membrane, the contact angle of the modified membrane decreases significantly, indicating that the hydrophilicity of the PVDF membrane after modification is improved. The contact angle reaches the minimum value at 30°C, the pure water flux of the modified PVDF membrane reaches the maximum value at 20°C, the influence of the post-treatment temperature on the contact angle of the membrane surface is not large, and the improvement of hydrophilicity is irregular. One possible explanation for this phenomenon is that this dopamine polymer structure contains a large number of hydrogen bonds, so as the temperature increases, it will also cause the polymer molecules to lose water, making the structure of the polymer more constricted, resulting in the membrane pores becoming rougher and the membrane surface becoming denser. Thus, the flux of pure water at higher temperature is reduced compared to that in room temperature.

### 3.2 Experimental results and analysis of the hydrophilic response surface

Based on the aforementioned single-factor optimal level, the values of solution concentration (A), coating time (B), and temperature (C) levels in the three-factor three-level response surface analysis experiment of this study are shown in Table 1.

**TABLE 1:** Response surface optimization experimental factor-level table.

Variable	Levels		
	−1	0	1
DA concentration (g/L)	1.0	1.5	2.0
Coating time (h)	4.0	5.0	6.0
Temperature (°C)	20	30	40

Design-Expert software was used to design the experimental factors and levels. Table 2 shows the hydrophilicity results under 17 different experimental conditions in the Box–Behnken experiment, and the hydrophilicity effect Y was used as the evaluation index (Jamalludin et al., 2018; Tibi et al., 2021). The smaller Y value means the smaller the contact angle of the modified PVDF membrane, the more significant hydrophilicity. The quadratic multinomial regression equation between the hydrophilicity of the modified membrane and each influencing factor established by the least-squares method is calculated according to the following equation.

$$Y=33.46-0.7875A+0.15B-0.4125C+AB+1.23AC-1.1BC+{4.81A}^{2}+0.3325B^{2}+{0.9075C}^{2},$$ (2)

where *Y* is the hydrophilicity °, *A* is the DA solution concentration g/L, *B* is the coating time h, and *C* is the temperature °C.

**TABLE 2:** Response surface optimization experimental scheme and results.

Run	DA concentration (g.L^-1^)	Coating time (h)	Temperature (°C)	Contact angle (°)
1	1.0	4	30	40.5
2	1.0	5	20	40.9
3	1.0	5	40	38.6
4	1.0	6	30	38.7
5	1.5	4	20	34.3
6	1.5	4	40	34.7
7	1.5	6	20	36.9
8	1.5	6	40	32.9
9	1.5	5	30	33.5
10	1.5	5	30	33.1
11	1.5	5	30	33.3
12	1.5	5	30	33.9
13	1.5	5	30	33.5
14	2.0	4	30	36.5
15	2.0	5	20	37.3
16	2.0	5	40	39.9
17	2.0	6	30	38.7

Table 3 shows the results of ANOVA of the aforementioned hydrophilic response surface experiment, the *p*-value reflects the degree of significance in which the experimental data are not relevant to the model; when the *p*-value is less than 0.01, it can be seen that the term has a strong significant effect on the response value, and when its value is less than 0.05, it indicates a high degree of significance, and *vice versa*. According to the results in the table, the *p*-value of A (*p* = 0.0084) is less than 0.01, indicating that the hydrophilic effect is more significant. The correction determination coefficient of the model is *R* ^2^ = 0.9795, indicating that this model can explain 97.95% of the response value change and fits well with the actual experiment (Vera Candioti et al., 2014; Shen et al., 2020; Li et al., 2023). CV % (coefficient of variation of Y) indicates the accuracy of the experiment. The higher the CV % value, the lower the reliability of the experiment, and the CV % = 1.69 of the experiment in this design is very low, indicating that the reliability of the experiment is very high. The aforementioned analysis shows that the model fits well, the experimental error is small, the model is suitable, and this model can be used to analyze and predict the change of hydrophilicity of PVDF/DA modified membranes.

**TABLE 3:** Regression model ANOVA table.

Source	Sum of squares	Df	Mean square	F-value	*p*-value
Model	126.13	9	14.01	37.24	<0.0001
A	4.96	1	4.96	13.18	0.0084
B	0.1800	1	0.1800	0.4783	0.5115
C	1.36	1	1.36	3.62	0.0989
AB	4.00	1	4.00	10.63	0.0139
AC	6.00	1	6.00	15.95	0.0052
BC	4.84	1	4.84	12.86	0.0089
A^2^	97.31	1	97.31	258.57	<0.0001
B^2^	0.4655	1	0.4655	1.24	0.3028
C^2^	3.47	1	3.47	9.21	0.0190
Residual	2.63	7	0.3764		
Lack of Fit	2.28	3	0.7608	8.65	0.0319
Pure Error	0.3520	4	0.0880		
Cor Total	128.77	16			

*R* ^2^ = 0.9795, AdjR^2^ = 0.9532, and CV% = 1.69.

### 3.3 Response surface interaction analysis

**FIGURE 4:**
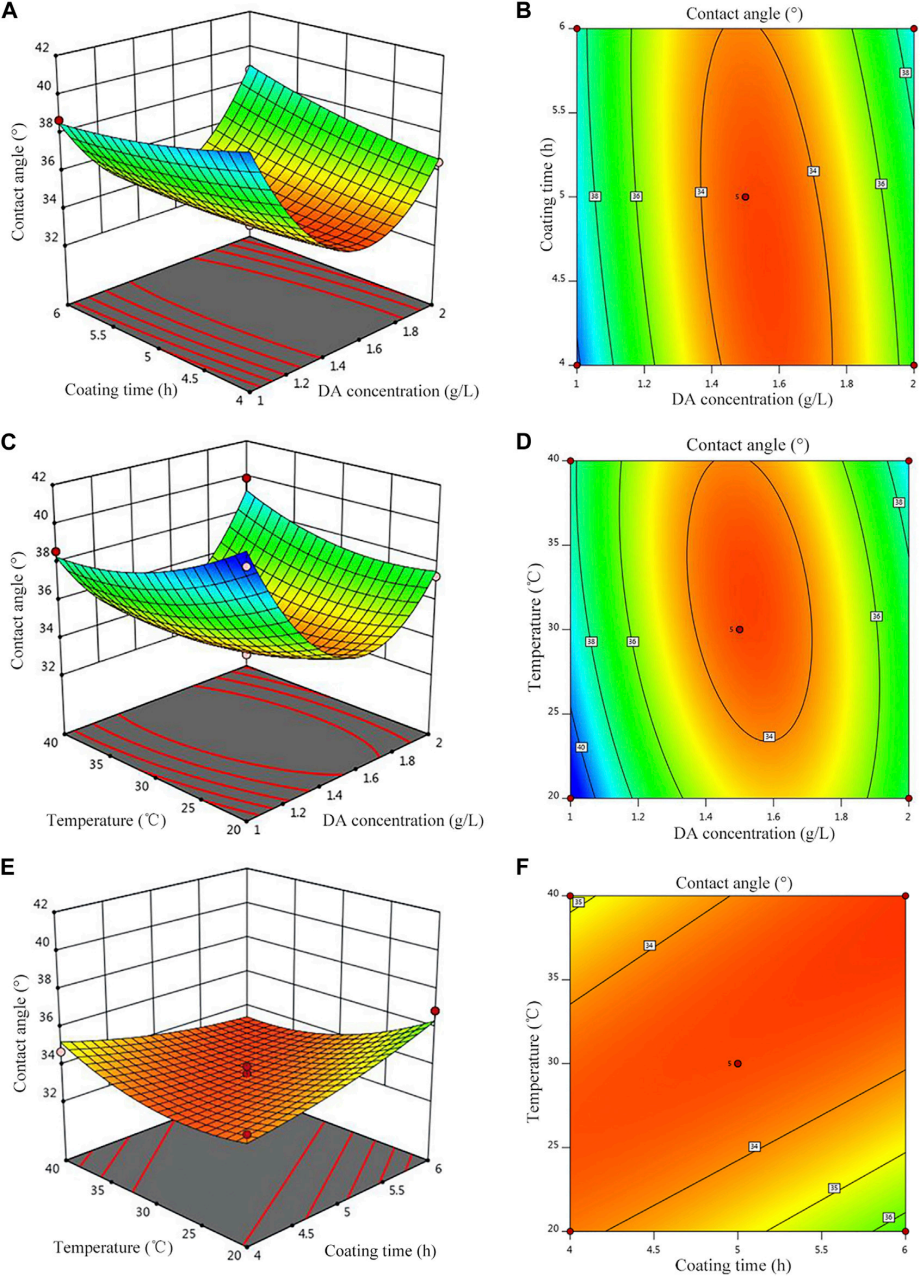
Effect of the parameters on the contact angle. **(A)** Contour of A and B, **(B)** 3D surfaces of A and B, **(C)** contour of A and C, **(D)** 3D surfaces of A and C, **(E)** Contour of B and C, and **(F)** 3D surfaces of B and C.

Figure 4 shows the response surface plot and contour plot corresponding to the regression equation. The greater the surface slope of the response surface plot, the more significant the effect of this factor on the response value. The contour plot can visually reflect the significance of the interaction between the two variables, indicating that the interaction of the two factors has no significant effect on the response value when the contour plot is circular, but it is significant when it is elliptical or saddle-shaped. It can be seen from Figures 4A, B that when the polymerization time is 4 h, with the increase of the concentration of the DA solution, the hydrophilicity first decreases and then increases; when the coating time is 6 h, the hydrophilicity decreases with the increase of the concentration of the DA solution and reaches a minimum value, and then, hydrophilicity increases with the increase of the concentration of the DA solution, and the surface inclination along the concentration of the DA solution is larger, indicating that the relative polymerization time and the influence of the DA solution concentration on hydrophilicity are more significant. Similarly, in Figures 4C, D, the concentration of the DA solution has a more significant effect on hydrophilicity than that of post-treatment temperature, and when the temperature is unchanged, with the increase of the DA solution concentration, hydrophilicity first decreases and then increases. In Figures 4E, F, hydrophilicity decreases with increasing treatment temperature when the coating time is constant but decreases with increasing coating time when the post-treatment temperature is constant.

### 3.4 Parameter optimization and validation

**FIGURE 5:**
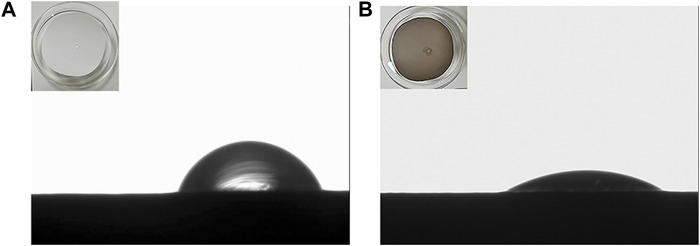
Contact angle images: **(A)** PVDF membrane and **(B)** PVDF/DA membrane.

Design-Expert software was used to optimize the hydrophilicity of PVDF/DA modified membranes, and the minimum value of hydrophilicity was taken as the target optimization value; the optimal conditions for the hydrophilicity of the modified membrane were as follows: the concentration of the DA solution was 1.65 g/L, the coating time was 4.45 h, and the temperature was 24.92°C, at which time the model predicted that the hydrophilicity of the modified membrane was 32.76°. Based on the aforementioned response surface experimental results, considering the feasibility of actual operation, the parameters of the modified membrane were corrected to the DA solution concentration 1.65 g/L, coating time 4.5 h, and temperature 25°C to test the reliability of the response surface method. The verification experiment was carried out under this condition, and the average value was repeated three times. The actual value of hydrophilicity of the modified membrane was measured using a contact angle goniometer to be 33.9°. The contact angles of the PVDF and PVDF/DA membranes are shown in Figure 5. The absolute value of the relative error between the actual value and the predicted value is only 3.36%, which is a reasonable margin of error. Under the experimental condition of 0.10 MPa, the average pure water flux of the PVDF/DA membrane within 60 min was 126.24 mL/(cm^2^. h). The results show that the established prediction model has good predictability and can predict the hydrophilicity of the PVDF/DA modified membrane based on actual needs.

### 3.5 Characterization of PVDF/DA modified membranes

**FIGURE 6:**
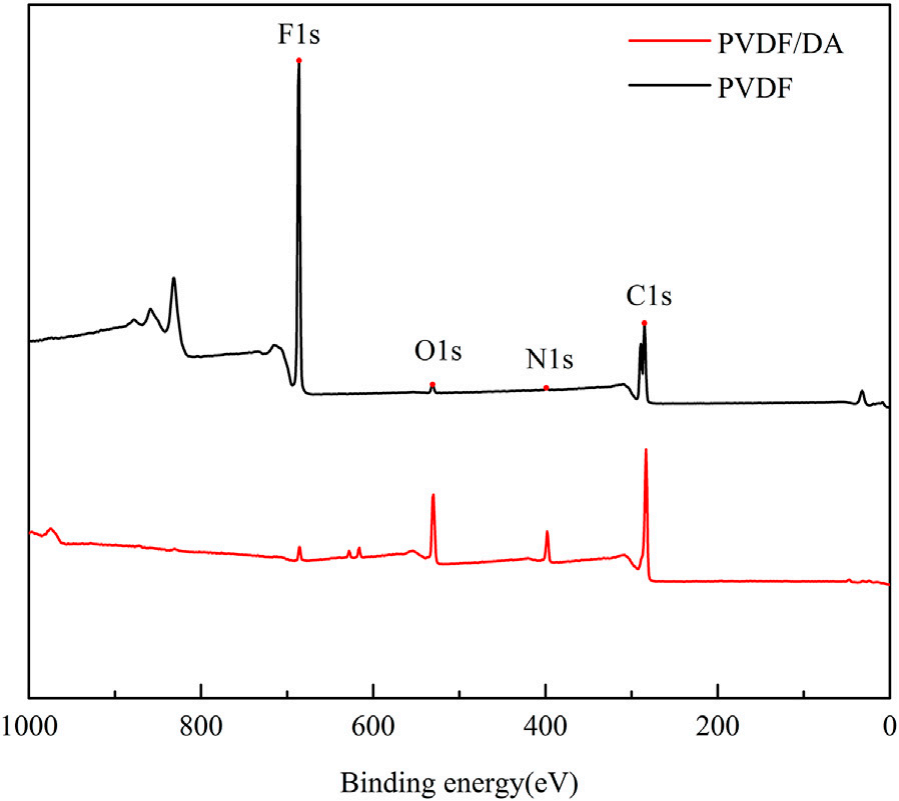
XPS full spectrum of PVDF and PVDF/DA membranes.

The surface compositions of the original and modified membranes were analyzed using XPS. The XPS spectra of the PVDF and PVDF/DA membranes are shown in Figure 6, from which it can be seen that the original PVDF membranes show distinct characteristic peaks, especially at 686 and 285 eV. These peaks are characteristic peaks for elements F and C, respectively. After DA modification, the characteristic peaks corresponding to O and N elements were found, and the C1s peak intensity was also enhanced, which was due to the presence of benzene rings on DA and containing amide and hydroxyl groups. It indicates that the polydopamine layer has been coated on the PVDF membrane. At the same time, the F1s peak intensity diminishes, indicating that the modified layer has a certain thickness, resulting in a smaller thickness of the base membrane detected by XPS.

**FIGURE 7:**
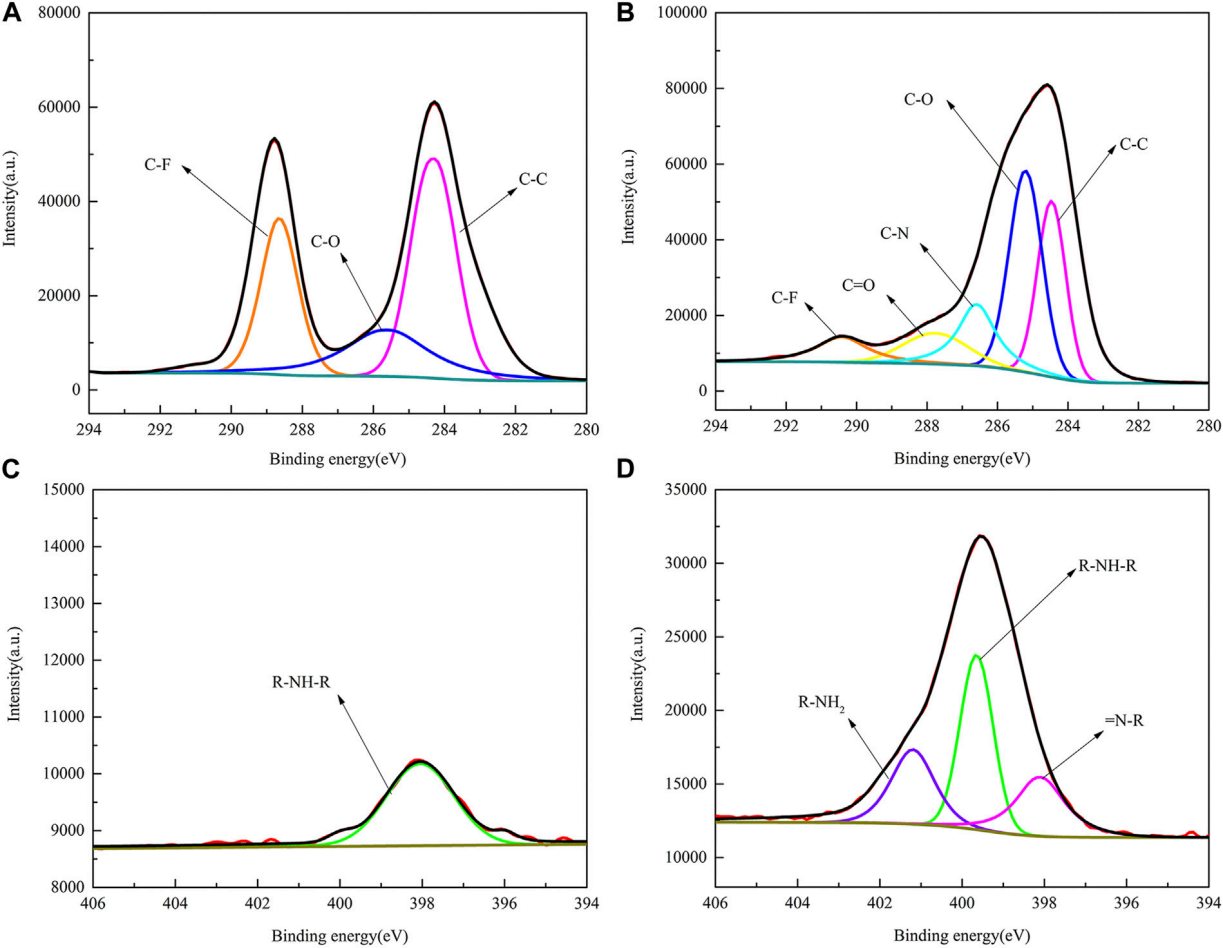
XPS fitting spectra of PVDF and PVDF/DA membranes. **(A)** PVDF-C1s, **(B)** PVDF/DA-C1s, **(C)** PVDF-N1s, and **(D)** PVDF/DA-N1s.

To further understand the process of dopamine-modified PVDF membranes, the XPS spectra of two elements, C and N, on the membrane surface were fitted by splitting the peaks, as shown in Figure 7. In Figure 7A, the Cls element spectrum can be fitted by splitting three different peaks, namely, C-C (284.7 eV), C-O (285.5 eV), and C-F (288.6 eV), while PVDF/DA in Figure 7B contains two new peaks, C-N (286.6 eV) and C=O (287.8 eV), indicating improved coverage of hydrophilic oxygen-containing groups on the membrane surface. In Figure 7C, the PVDF membrane is fitted with only one peak, R-NH-R. The elemental peaks of N1s in Figure 7D can be fitted to three different peaks: R-NH^2^, R-NH-R, and = N-R. The XPS analysis results further indicate that the polydopamine layer has been successfully coated onto the PVDF membrane.

**FIGURE 8:**
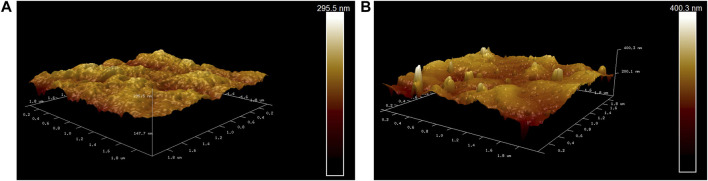
AFM images: **(A)** PVDF membrane and **(B)** PVDF/DA membrane.

Figures 8A, B show atomic force microscopic (AFM) images of two membranes. There are some relatively uniform small tumor structures on the surface of the PVDF membrane, but the bright peaks on the surface of the PVDF/DA modified membrane increase significantly. Compared with the original membrane, the average roughness increased from 15.9 to 22 nm, an increase of 1.38 times, which indicates that there is a polydopamine coating on the surface of the modified membrane. The maximum roughness of the PVDF membrane is 153 nm, and the maximum roughness of the PVDF/DA modified membrane is 371 nm. It was shown that the polydopamine layer aggravated the degree of unevenness on the membrane surface, increasing the hydrophilicity of the membrane.

**FIGURE 9:**
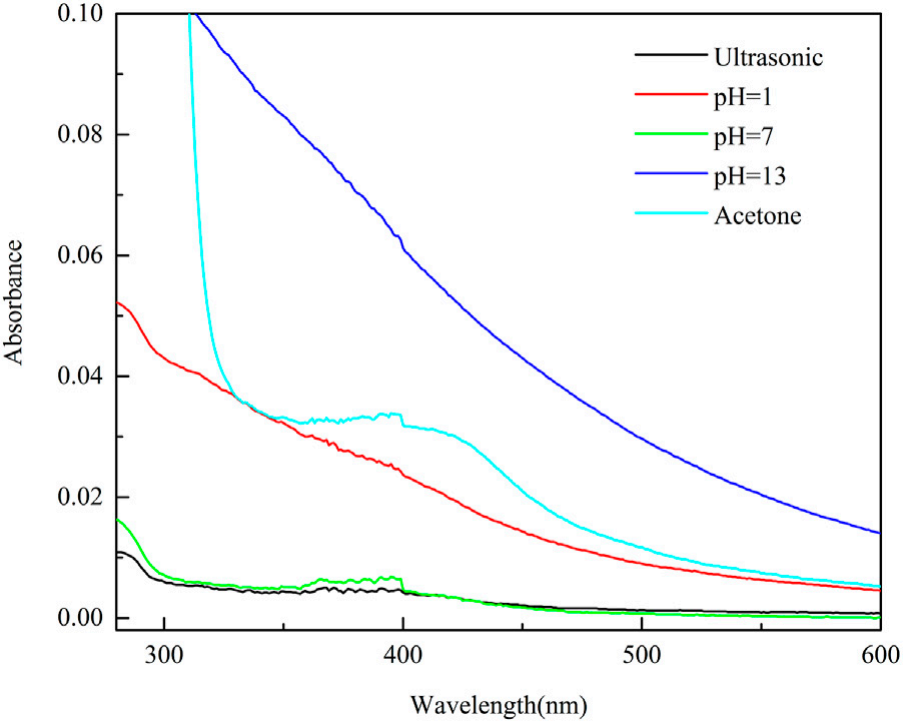
UV-Vis spectra of eluates from PVDF/DA membranes in different environments.

Figure 9 shows that the PVDF/DA membrane has good stability under ultrasonic, acidic, and neutral conditions, and the absorbance value is low, indicating that the dissolution and elution of the polymer layer is small, and the polydopamine layer is more stable. However, in the solution of a strong organic solvent and strong base, the absorbance value is relatively large, indicating that the degree of damage to the polydopamine layer is greater. Generally, the pH range of wastewater treated in MBR is between 6 and 9, so PVDF/DA membranes can be used in MBR and help in maintaining certain stability.

### 3.6 MBR parallel comparison experiment

The COD removal rate in the PVDF/DA modified membrane reactor was 95.74%, which was higher than the removal rate of 90.43% in the PVDF membrane reactor, and the COD removal rate per unit membrane area of the PVDF/DA modified membrane was found to be 4.64 mg/(L.cm^2^), which was 1.65 times higher than that of the PVDF membrane. As shown in Table 4, the results indicated that the PVDF/DA membrane has a stronger COD removal capacity relative to that of the pure PVDF membrane, particularly relying on the degradation of microorganisms in the reactor and the interception of membranes. This further demonstrates the promising application and capability of this membrane modified by dopamine in MBR.

**TABLE 4:** Comparison of COD removal experiments before and after modification.

Membrane	COD (inlet) (mg.L^-1^)	COD (outlet) (mg.L^-1^)	Effective treatment area (mg. (L.cm^2^)^−1^)
PVDF membrane	178.84	17.97	2.81
PVDF/DA membrane	293.48	12.49	4.64

The cultured mature biofilm was scraped off the membrane surface, three parallel samples were taken as the average, and the sum of protein, polysaccharide, and DNA content was used as the EPS content. Compared with PVDF/DA modified membranes, the total amount of EPS of PVDF membranes increased by 1.46 times, of which proteins, polysaccharides, and DNA increased by 1.33, 1.56, and 1.29 times, respectively, as shown in Table 5. Proteins combine with the membrane surface by dipole action or colloidal formation gel due to their amphoteric characteristics, but after DA surface modification, the adsorption capacity of protein molecules decreases. The content of polysaccharides is relatively large because polysaccharide molecules are easy to form gels and easy to form a filter cake layer on the membrane surface. The EPS content can indirectly indicate the degree of membrane fouling, which shows that DA modification effectively slows down membrane fouling.

**TABLE 5:** EPS composition analysis of PVDF membranes and PVDF/DA membranes.

Membrane	Protein (mg·g^-1^VSS)	Polysaccharide (mg·g^-1^VSS)	DNA (mg·g^-1^VSS)	Total EPS (mg·g^-1^VSS)
PVDF membrane	6.08	12.09	0.98	19.15
PVDF/DA membrane	4.58	7.77	0.76	13.11

### 3.7 Alpha diversity analysis

**FIGURE 10:**
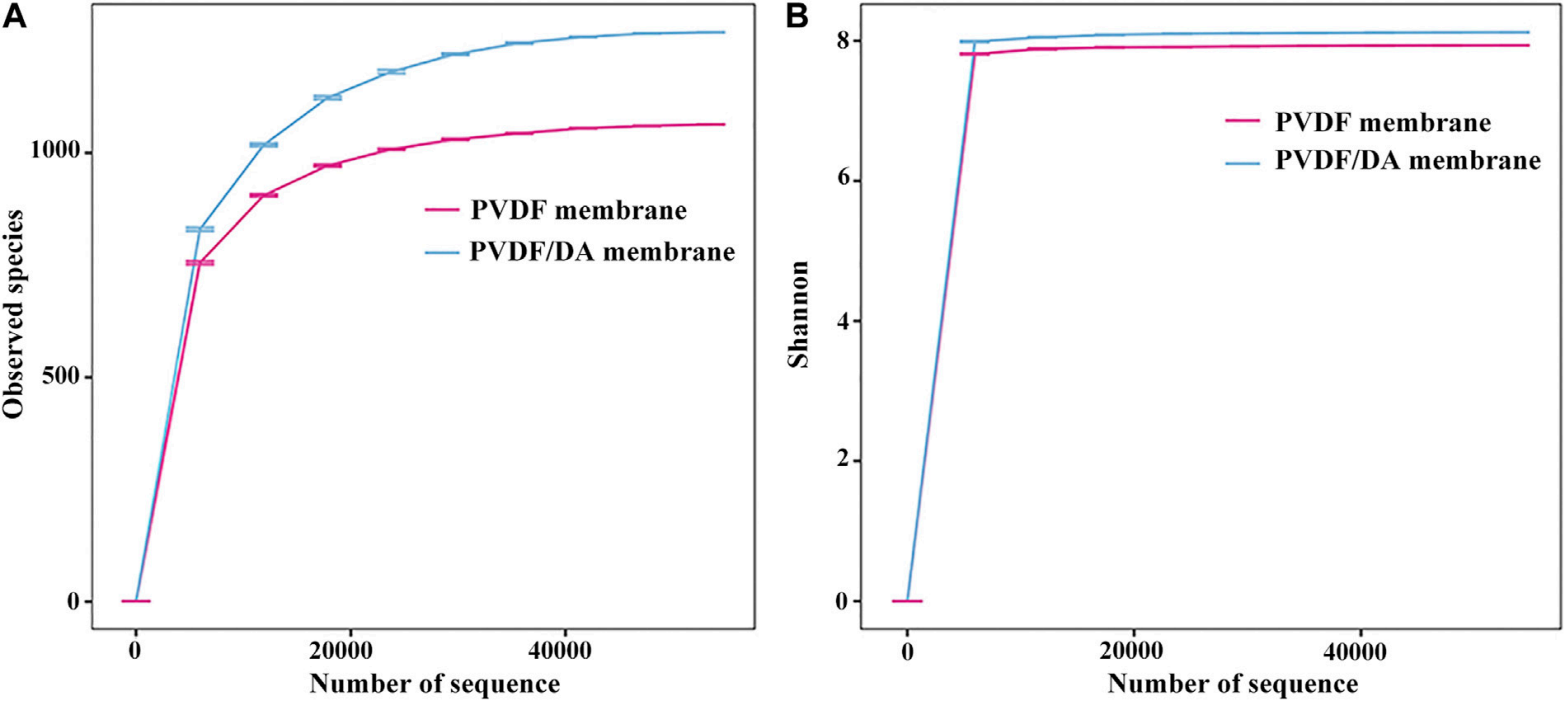
Index dilution curve: **(A)** observed species and **(B)** Shannon index.

Alpha diversity analysis refers to the diversity in a specific environment or ecosystem, particularly reflecting the two influencing factors of species composition richness and uniformity, and results are shown in Figure 10.

In this experiment, the observed species index and Shannon index were selected for plot analysis. Figure 10A shows the observed species index dilution curve of each sample. The abscissa is the number of sampling sequences in the sample, and the ordinate is the number of different species in the sample. It can be found that the sample curves tend to be flat, indicating that the number of sequences in the experiment is sufficient. Figure 10A shows that the number of species detected on PVDF/DA membranes is greater than that of PVDF membranes. Figure 10B shows the dilution curve of the Shannon index; the larger the Shannon index, the greater the uncertainty, indicating that there are more unknown factors in this community, that is, high diversity. On one hand, this is because the modified PVDF membrane is more favorable for the growth of biofilms in terms of hydrophilicity and surface roughness; on the other hand, it may be because the dopamine surface is biocompatible and has strong adhesion compared to the PVDF membrane, which promotes the attachment and growth of microorganisms on the dopamine surface.

## 4 Conclusion

In this study, we successfully increased the hydrophilicity of the PVDF membrane from 69° to 33.9° using the response surface method. The model can predict the results more accurately within the experimental range, and the absolute relative error between the actual value and the predicted value is only 3.36%. Through the determination of COD, EPS content, and alpha diversity analysis, it was found that the PVDF/DA modified membrane had a good degradation effect, antifouling performance, and biodiversity. At the same time, the accuracy and scientific validity of the response surface method for the PVDF/DA modified membrane are verified, which provides a reference for subsequent evaluation and application.

## Data Availability

The original contributions presented in the study are included in the article/Supplementary Material; further inquiries can be directed to the corresponding authors.

## References

[B1] Al Mayyahi A. (2018). Important approaches to enhance reverse osmosis (RO) thin film composite (TFC) membranes performance. Membr. (Basel) 8 (3), 68. 10.3390/membranes8030068 PMC6161033 30134581

[B2] Ayyaru S. Ahn Y. (2017). Application of sulfonic acid group functionalized graphene oxide to improve hydrophilicity, permeability, and antifouling of PVDF nanocomposite ultrafiltration membranes. J. Membr. Sci. 525, 210–219. 10.1016/j.memsci.2016.10.048

[B3] Cheng X. Q. Zhang C. Wang Z. X. Shao L. (2016). Tailoring nanofiltration membrane performance for highly-efficient antibiotics removal by mussel-inspired modification. J. Membr. Sci. 499, 326–334. 10.1016/j.memsci.2015.10.060

[B4] Dong L. Liu X. Xiong Z. Sheng D. Zhou Y. Lin C. (2018). Design of UV-absorbing PVDF membrane via surface-initiated AGET ATRP. Appl. Surf. Sci. 435, 680–686. 10.1016/j.apsusc.2017.11.135

[B5] Drews A. (2010). Membrane fouling in membrane bioreactors—characterisation, contradictions, cause and cures. J. Membr. Sci. 363 (1), 1–28. 10.1016/j.memsci.2010.06.046

[B6] Guo H. Deng Y. Yao Z. Yang Z. Wang J. Lin C. (2017). A highly selective surface coating for enhanced membrane rejection of endocrine disrupting compounds: Mechanistic insights and implications. Water Res. 121, 197–203. 10.1016/j.watres.2017.05.037 28535433

[B7] Huang Q. Chen J. Liu M. Huang H. Zhang X. Wei Y. (2020). Polydopamine-based functional materials and their applications in energy, en-vironmental, and catalytic fields State-of-the-art review. Chem. Eng. J. 387, 124019. 10.1016/j.cej.2020.124019

[B8] Jamalludin M. R. Harun Z. Hubadillah S. K. Dzarfan Othman M. H. Kamarudin N. H. Yunos M. Z. (2018). Optimization of polysulfone/graphene oxide/polyethylene glycol/triaminopyrimidine by using response surface methodology. Mater. Sci. Eng. 318 (1), 012064. 10.1088/1757-899x/318/1/012064

[B9] Lee H. Dellatore S. M. Miller W. M. Messersmith P. B. (2007). Mussel-inspired surface chemistry for multifunctional coatings. Science 318 (5849), 426–430. 10.1126/science.1147241 17947576 PMC2601629

[B10] Li G. Liu B. Bai L. Shi Z. Tang X. Wang J. (2020). Improving the performance of loose nanofiltration membranes by poly-dopamine/zwitterionic polymer coating with hydroxyl radical activation. Sep. Purif. Technol. 238, 116412. 10.1016/j.seppur.2019.116412

[B11] Li T. Su T. Wang J. Zhu S. Zhang Y. Geng Z. (2023). Simultaneous removal of sulfate and nitrate from real high-salt flue gas wastewater concentrate via a waste heat crystallization route. J. Clean. Prod. 382, 135262. 10.1016/j.jclepro.2022.135262

[B12] Meng F. Zhang S. Oh Y. Zhou Z. Shin H. S. Chae S. R. (2017). Fouling in membrane bioreactors: An updated review. Water Res. 114, 151–180. 10.1016/j.watres.2017.02.006 28237783

[B13] Muchtar S. Wahab M. Y. Mulyati S. Arahman N. Riza M. (2019). Superior fouling resistant PVDF membrane with enhanced filtration performance fabricated by combined blending and the self-polymerization approach of dopamine. J. Water Process Eng. 28, 293–299. 10.1016/j.jwpe.2019.02.012

[B14] Mulyati S. Muchtar S. Arahman N. Syamsuddin Y. Mat N. N. Yub H. N. (2020). Two-step dopamine-to-polydopamine modification of polyethersulfone ultrafiltration membrane for enhancing anti-fouling and ultraviolet resistant properties. Polym. (Basel) 12 (9), 2051. 10.3390/polym12092051 PMC7569805 32916778

[B15] Qiu W. Z. Yang H. C. Xu Z. K. (2018). Dopamine-assisted co-deposition: An emerging and promising strategy for surface modification. Adv. Colloid Interface Sci. 256, 111–125. 10.1016/j.cis.2018.04.011 29776584

[B16] Shen C. Yang J. Cui Z. Qin S. Qin Q. (2020). The investigation of hydrophilic modification of membrane surface based on the mono-esterification between maleic anhydride and polyethylene glycol: Response surface methodology, reaction kinetics and performance analysis. J. Taiwan Inst. Chem. E 113, 193–201. 10.1016/j.jtice.2020.07.002

[B17] Su X. Zhang Z. (2018). Structural characteristics of extracellular polymeric substances (EPS) in membrane bioreactor and their adsorptive fouling. Water Sci. Technol. 77 (5-6), 1537–1546. 10.2166/wst.2018.033 29595156

[B18] Sun C. Feng X. (2017). Enhancing the performance of PVDF membranes by hydrophilic surface modification via amine treatment. Sep. Purif. Technol. 185, 94–102. 10.1016/j.seppur.2017.05.022

[B19] Sun W. Liu J. Chu H. Dong B. (2013). Pretreatment and membrane hydrophilic modification to reduce membrane fouling. Membranes 3 (3), 226–241. 10.3390/membranes3030226 24956947 PMC4021944

[B20] Tang Y. Sun J. Li S. Ran Z. Xiang Y. (2019). Effect of ethanol in the coagulation bath on the structure and performance of PVDF-g-PEGMA/PVDF membrane. J. Appl. Polym. Sci. 136 (17), 47380. 10.1002/app.47380

[B21] Tibi F. Park S. J. Kim J. (2021). Improvement of membrane distillation using PVDF membrane incorporated with TiO(_2_) modified by silane and optimization of fabricating conditions. Membr. (Basel) 11 (2), 95. 10.3390/membranes11020095 PMC7912162 33572959

[B22] Vera Candioti L. De Zan M. M. Cámara M. S. Goicoechea H. C. (2014). Experimental design and multiple response optimization. Using the desirability function in analytical methods development. Talanta 124, 123–138. 10.1016/j.talanta.2014.01.034 24767454

[B23] Wang J. Zhang S. Wu P. Shi W. Wang Z. Hu Y. (2019). *In situ* surface modification of thin-film composite polyamide membrane with zwitterions for enhanced chlorine resistance and transport properties. ACS Appl. Mater Interfaces 11 (12), 12043–12052. 10.1021/acsami.8b21572 30817111

[B24] Wang Y. Sun T. Tong L. Gao Y. Zhang H. Zhang Y. (2023). Non-free Fe dominated PMS activation for enhancing electro-Fenton efficiency in neutral wastewater. J. Electroanal. Chem. 928, 117062. 10.1016/j.jelechem.2022.117062

[B25] Wang Z. Yang H. He F. Peng S. Li Y. Shao L. (2019). Mussel-Inspired surface engineering for water-remediation materials. Matter-US 1, 115–155. 10.1016/j.matt.2019.05.002

[B26] Xiang Y. Liu F. Xue L. (2015). Under seawater superoleophobic PVDF membrane inspired by polydopamine for efficient oil seawater separation. J. Membr. Sci. 476, 321–329. 10.1016/j.memsci.2014.11.052

[B27] Xiao K. Liang S. Wang X. Chen C. Huang X. (2019). Current state and challenges of full-scale membrane bioreactor applications: A critical review. Bioresour. Technol. 271, 473–481. 10.1016/j.biortech.2018.09.061 30245197

[B28] Xue Q. Cao H. Meng F. Quan M. Gong Y. (2017). Cell membrane mimetic coating immobilized by mussel-inspired adhesion on commercial ultrafiltration membrane to enhance antifouling performance. J. Membr. Sci. 528, 1–11. 10.1016/j.memsci.2017.01.009

[B29] Yang X. Yan L. Wu Y. Liu Y. Shao L. (2019). Biomimetic hydrophilization engineering on membrane surface for highly-efficient water purification. J. Membr. Sci. 589, 117223. 10.1016/j.memsci.2019.117223

[B30] Yu C. Dongxu L. Hongyu C. Suiyi Z. Xianze W. Jiakuan Y. (2022). Review of resource utilization of Fe-rich sludges: Purification, upcycling, and application in wastewater treatment. Environ. Rev. 30 (3), 460–484. 10.1139/er-2021-0038

[B31] Zeng G. He Y. Yu Z. Zhan Y. Ma L. Zhang L. (2016). Preparation and characterization of a novel PVDF ultrafiltration membrane by blending with TiO_2_-HNTs nanocomposites. Appl. Surf. Sci. 371, 624–632. 10.1016/j.apsusc.2016.02.211

[B32] Zhang H. Hu Q. Zheng X. Yin Y. Wu H. Jiang Z. (2019). Incorporating phosphoric acid-functionalized polydopamine into Nafion polymer by *in situ* sol-gel method for enhanced proton conductivity. J. Membr. Sci. 570-571, 236–244. 10.1016/j.memsci.2018.10.021

[B33] Zhang L. Li Y. Guo J. Kan Z. Jia Y. (2023). Catalytic ozonation mechanisms of Norfloxacin using Cu-CuFe_2_O_4_ . Environ. Res. 216, 114521. 10.1016/j.envres.2022.114521 36216118

[B34] Zhao X. Xuan H. Chen Y. He C. (2015). Preparation and characterization of superior antifouling PVDF membrane with extremely ordered and hydrophilic surface layer. J. Membr. Sci. 494, 48–56. 10.1016/j.memsci.2015.07.052

[B35] Zin G. Wu J. Rezzadori K. Petrus J. C. C. Di Luccio M. Li Q. (2019). Modification of hydrophobic commercial PVDF microfiltration membranes into superhydrophilic membranes by the mussel-inspired method with dopamine and polyethyleneimine. Sep. Purif. Technol. 212, 641–649. 10.1016/j.seppur.2018.10.014

